# Supplementary material for: Sex Differences in Plasma MicroRNA Biomarkers of Early and Complicated Diabetes Mellitus in Israeli Arab and Jewish Patients
Source: Noncoding RNA. 2019 Apr 5;5(2):32. doi: 10.3390/ncrna5020032 (PMC6631160; doi:10.3390/ncrna5020032)
Supplement: Supplementary file 1 [file ncrna-05-00032-s001.pdf]

Supplemental Table S1

	A<br>Healthy controls<br>(n=30)	B<br>Early DM<br>(n=29)	C<br>Complicated DM with PVD + Retinopathy<br>(n=29)	p-value
Age (years)	31 ± 11	64 ± 10	65 ± 10	0.001
Sex, M/F	50%/50%	62%/38%	65%/35%	0.4
Abd. girth (cm)	86 ± 12	100 ± 4	100 ± 7	0.001
BMI (kg/m^2)	25 ± 4	30 ± 5	30 ± 7	0.001
SBP (mm Hg)	113 ± 8	141 ± 20	142 ± 30	0.001
DBP (mmHg)	69 ± 7	76 ± 8	74 ± 12	0.007
Glucose (mM)	91 ± 10	167 ± 83	179 ± 84	0.001
TG (mg/dl)	123 ± 89	140 ± 73	175 ± 115	0.01
LDL (mg/dl)	112 ± 35	95 ± 64	94 ± 44	0.01
HDL (mg/dl)	74 ± 13	31 ± 12	35 ± 10	0.001
HbA1C (%)	5.1 ± 0.3	7.7 ± 1.9	8.6 ± 2.0	0.001
HbA1C (mmol/mol)	32 ± 4	61 ± 20	70 ± 22	
U-Prot (mg/dl)	0	203 ± 56	958 ± 411	N/A
**Ethnic/religious affiliation**				total
**Muslim Arab**	15	17	19	51
**Christian Arab**	5		4	9
**Jew**	5	9	6	20
**Druse**	6			6
**Undetermined ethnicity**		2		2

Supplemental Table S1: Clinical measurements and ethnic/religious affiliations of the participant groups. P-value: t-test between groups A and B+C. Acronyms: BMI – body mass index; SBP – systolic blood pressure; DBP – diastolic blood pressure; TG – triglycerides; LDL – low density lipoprotein; HDL – high density lipoprotein; HbA1C – Glycated hemoglobin; U-Prot: urine protein excretion.

Supplemental Table S2

miRNA	B	CI 95%	SE	T	p
**using plasma miRNA levels only**					
Step 1: miR-146a-5p	0.44	0.3	0.15	2.95	0.004
Step 2: miR-16-2-3p	0.12	0.10	0.05	2.17	0.03
Step 3: miR-126-5p	-0.22	0.2	0.05	-2.5	0.01
Step 4: miR-30d	-0.40	0.3	0	-2.8	0.005
**using plasma miRNA levels and clinical measurements**					
miR-423	13	0.7	3.6	3.7	0.0004

Supplemental Table S2: Multiple stepwise regression analysis to distinguish early vs. complicated DM using a panel of plasma miRNAs. **B** - regression coefficient; CI 95% - confidence interval of 95%; **SE** - standard errors of the regression coefficient; **T** - quotient of the coefficient; **p** - p-value (two-sided t-test).
